# NEWS

**DOI:** 10.7189/jogh.04.020202

**Published:** 2014-12

## BILL AND MELINDA GATES FOUNDATION

➢ The BMGF is set to expand its presence in Ethiopia and Nigeria in 2014, and may open new offices in East and West Africa. The Foundation emphasised that it does not intend to become a large field operating agency, despite its recent rapid growth, and tries to strike a balance between having staff based in Africa away from its Seattle headquarters, and the need to keep a presence in country offices to link between governments and more traditional donors. It has formed “leveraged partnerships” with the Ethiopian government to develop a strategy for agricultural investment, aligning donor funding in a more unified fashion, and believes that this model can work elsewhere. (*Devex*, 7 Jul 2014)

➢ The BMGF has awarded US$ 25 million to Oregon Health and Science University, who are developing a vaccine that shows promise in preventing HIV infection in primates. The scientists hope to develop a vaccine that prevents infection in exposed people, and eliminates it from those already infected. The grant funding will establish if the vaccine can be safely used in humans in a clinical trial, and to develop a version suitable for larger–scale testing, which is needed to bring it to market. It is expected that this will take at least 10 years. (*Reuters*, 3 Sep 2014)

➢ The BMGF announced that it is accepting applications for Round 14 of the Grand Challenges Explorations Initiative, which “seeks innovative solutions to some of the world’s most pressing global health and development problems” with US$ 100 000 grants available to early stage, innovative ideas. The topics for this round are: enabling universal acceptance of mobile money payments; reducing childhood deaths from pneumonia; reducing malaria transmission from outdoor mosquitoes; supporting new mosquito–control approaches; measuring brain development and gestational age; and integrating community–based interventions. (*BMGF*, 8 Sep 2014)

➢ The BMGF pledged US$ 50 million to the fight against Ebola in West Africa, the largest sum it has ever committed to one outbreak. This is in addition to the US$ 12 million support it has already given to the WHO, UNICEF and Centers for Disease Control and Prevention to support efforts against Ebola. The new funding will be used for emergency operations and to help develop drugs, diagnostics and vaccines. The BMGF could make funds immediately available, unlike other donors. The UN estimated that defeating this outbreak could cost at least US$ 600 million. (*New York Times*, 10 Sep 2014)

➢ At an event commemorating the 10th anniversary of the Grand Challenges Initiative, a group of partners announced three new initiatives aimed at creating breakthroughs in science, namely: All Children Thriving; Putting Women and Girls at the Centre of Development; and Creating New Interventions for Global Health. “Melinda and I have always believed that advances in science can help reduce inequality in a big way. But you have to be willing to take some risks and see some projects fail. That’s the idea behind Grand Challenges – to focus bright scientists on the problems of the poorest, take some risks and delivery results. We’re delighted with what’s happened in the first decade, but we’re not satisfied, and we hope to see even more progress in the coming years,” says Bill Gates. (*BMGF*, 7 Oct 2014)

## GAVI ALLIANCE

➢ GAVI approved the strategic framework that would support a fully–funded Alliance to enable developing countries to immunise an additional 300 million children from 2016–2020, helping save an estimated 5–6 million lives. GAVI will consolidate progress on the rapid acceleration of GAVI–supported vaccine introductions, putting the world’s poorest countries on target to immunise almost 250 million children from 2011–2015, saving nearly 4 million lives. The new framework will bring the GAVI partners together to work towards these key goals: accelerate uptake and coverage of vaccines; increase effectiveness and efficiency of immunisation delivery; improve sustainability of national programmes; and shape markets for vaccines and other immunisation products. (*GAVI*, 19 Jun 2014)

➢ Niger has introduced vaccines against pneumococcal disease and rotavirus that should significantly reduce child mortality. Nearly a million children will be vaccinated against the diseases that are the leading cause of child mortality in Niger, accounting for more than 30% deaths in children under 5 years. Niger joins Ghana, Tanzania and Burkina Faso in introducing the vaccines with GAVI support. This helps Niger play its part in the Global Action Plan for the Prevention and Control of Pneumonia and Diarrhoea, which aims to eliminate childhood deaths from these diseases by 2025. (*allafrica.com*, 5 Aug 2014)

➢ GAVI has given Uganda a grant of US$ 190 000 to support the country’s immunisation programmes. It will be used to buy fridges and motorcycles, support outreach programmes in inaccessible areas, improve immunisation data management, and train health workers in integrated disease surveillance and response. The government will also undertake a house–to–house polio campaign, targeting all children under 5 years. Although Uganda has not reported any polio cases since 2010, it is still vulnerable due to the influx of Somali refugees and its location within the wild polio importation belt (a band of countries at risk of infections from northern Nigeria). In response, Uganda will introduce injectable polio vaccinations in 2015, as part of the global eradication strategy. The funds will also be used to roll out the HPV vaccine to protect against cervical cancer. (*The Observer Uganda*, 12 Oct 2014)

➢ GAVI released the findings of an audit into US$ 29 million of funding given to Nigeria between 2011 to 2013. It found the Nigerian Federal Ministry of Health and the National Primary Health Care Development agency guilty of arrant malpractice and fraud, and the money – intended for vaccine procurement and health systems strengthening for children – badly mis–used. As a result, GAVI has suspended cash–based support from April 2014 onwards, and unused disbursed funds have been frozen. Weak leadership, disorganisation and conspiracy to commit fraud were all part of the malpractice, with lack of controls around procurement and evidence of collusion leading to irregular activities. The immediate past Minister of Health, Prof. Onyebuchi Chukwu acknowledged financial management weaknesses, gave assurances that the government would refund misappropriated money, and improve financial transparency and accountability. GAVI and the government will appoint an agent to oversee the management of GAVI’s grants to Nigeria. (*Sahara Reporters*, 30 Oct 2014)

➢ For the World Pneumonia Day on 12 Nov, GAVI published figures on vaccination against the leading causes of pneumonia. They show than almost 40 million children in developing countries received the pentavalent vaccine, and 15 million children were immunised with pneumococcal conjugate vaccine (PCV) in 2013 alone. Causing 1 million deaths a year amongst children aged under 5 years – mostly in developing countries – pneumonia is a leading cause of death. GAVI has supported the introduction of pentavalent vaccine in 73 of the world’s poorest countries, and funded PCV introduction in almost 45 countries. Children die from pneumonia due to lack of access to effective interventions, and vaccination, proper nutrition, hand–washing with soap, low–emission cooking stoves and exclusive newborn breastfeeding can help protect children against pneumonia. (*GAVI*, 12 Nov 2014)

## WORLD BANK

➢ The World Bank’s *Global Economic Prospects* shows developing countries facing disappointing growth in 2014, as adverse weather in the US, the Ukrainian crisis, rebalancing in China, political tensions in several countries, slow structural reforms and capacity constraints contribute to sub–5% growth. This is considered insufficient for job creation levels required to improve the lives of the poorest 40%. High–income countries’ recovery is accelerating, and overall the global economy will expand by 2.8% in 2014, 3.4% in 2015 and 3.5% in 2016. High–income countries will contribute 50% towards global growth, compared to sub–40% in 2013. Overall, growth is expected to be moderate in East Asia and the Pacific; recover modestly in the developing countries of Europe and Central Asia (although the situation in Ukraine is expected to cause a 1% decrease); continue weakly in Latin America and the Caribbean; gradually strengthen in the Middle East and North Africa; be subdued in South Asia; and strengthen in sub–Saharan Africa. (*World Bank*, 10 Jun 2014).

➢ The World Bank, World Health Organisation, Merck and other partners celebrated 40 years of success in controlling river blindness in Africa, via the African Programme for Onchocerciasis Control (APOC). APOC, one of the most successful public–private partnerships in Africa, treats 100 million people each year in 31 countries, using free medicines donated by Merck. The celebratory event highlighted lessons from APOC’s efforts to control river blindness and the impact of its programmes to neglected tropical diseases’ control and elimination. (*endtheneglect.org*, 19 Jun 2014)

➢ According to the World Bank, Egypt, Iran, Jordan, Lebanon, Libya, Tunisia and Yemen are trapped in a “poor policy–poor growth” cycle, preventing their economies from moving to a sustainable growth pattern, and worsening since the 2011 uprisings in the region. Each country has high unemployment rate with large numbers working in the informal sector, facing the related job insecurity and poor wages. Although economic growth has rebounded in Egypt and Tunisia, at current levels it cannot create enough jobs to absorb population increases. The Bank estimates that growth would need to double to reduce unemployment. Moreover, the ongoing instability in the region will affect short–term prospects. Each country has suffered from over–optimistic growth forecasts, leading to a lack of government action on the necessary reforms to ensure the private sector can drive growth and create jobs. (*World Bank*, 7 Aug 2014)

➢ The World Bank plans to raise up to US$ 500 million of Islamic bonds (‘sukuk’) to help fund immunisation. The International Finance Facility for Immunisation (IFFIm) will help issue these bonds, which would roll–forward future donor pledges into cash–in–hand today to finance immunisation efforts. As they are not interest–based, they follow Islamic religious principles banning charging interest and monetary speculation. The World Bank is increasingly considering using sukuk in other ways, eg, as ‘green bonds’ to finance energy–efficiency projects. This could help close the gap between ethics and investing in the region. (*Reuters*, 3 Sep 2014)

➢ The IMF and World Bank have pledged US$ 300 million in emergency aid to Guinea, Liberia and Sierra Leone, the countries most affected by the rapidly spreading Ebola virus. The IMF will provide US$ 127 million to cover the US$ 300 million financing gap, and will discuss further support in October. The World Bank has separately pledged US$ 200 million in emergency assistance, and the Bank’s President, Dr Jim Yong Kim, said that member countries were ready to provide funds. These measures were announced the day after US President Barack Obama confirmed that 3000 soldiers would be dispatched to help contain the outbreak. The WHO has warned is September that the number of deaths may double every three weeks. (*Financial Times*, 17 Sep 2014)

## UNAIDS/GLOBAL FUND

➢ The Global Fund has entered into new agreements with suppliers of artemisinin–based combination therapy (ACT) for malaria. It aims to improve value for money and save more lives, via maximised transition funding for a private sector co–payment for ACTs (the mechanism for providing high–quality medicines at reduced prices to low–income countries). The agreement will save US$ 100 million over two years, and more intelligent procurement will benefit overall global health efforts. (*Global Fund*, 10 Jun 2014)

➢ According to the UN, new HIV infections and AIDS–related deaths are declining, making it possible to control the HIV epidemic by 2030, and eventually end it. New infections fell by 38% from 2001, AIDS–related deaths fell by 35% from their 2005 peak, and globally the numbers of infections is stabilising at 35 million. According to UNAIDS, US$ 19.1 billion was spent on the HIV/AIDS response in 2013, and estimates that US$ 22–24 billion is needed in 2015. If the epidemic is controlled by 2030, 18 million new infections and 11.2 million AIDS–related deaths would be averted. Following evidence on the impact of early treatment on reducing the spread of HIV, the WHO set new guidelines which increased the numbers of people requiring treatment by 10 million. However, Médecins Sans Frontières argue 50% of people with HIV do not receive the treatment they need. (*Reuters*, 16 Jul 2014)

➢ The 20th International AIDS Conference in Melbourne, Australia, was in mourning at the loss of colleagues on the Malaysia Airlines flight when the plane, *en route* to the conference, was shot down over Ukraine. The International AIDS Society confirmed its lost colleagues include Joep Lange, pioneer of cheap anti–retrovirals for poor people, Pim de Kuijer from STOPAIDSNOW, Lucie van Mens and Maria Adriana de Schutter from AIDS Action Europe, WHO official Glenn Thomas and Jacqueline van Tongeren from the Amsterdam Institute for Global Health and Development. “We will honour their commitment and keep them in our hearts as we begin our programmes on Sunday,” said the Society’s President, Francoise Barre–Sinoussi. (*yahoo.com*, 18 Jul 2014)

➢ The UNAIDS *Gap Report* reveals that scaling–up antiretroviral therapy (ART) access to HIV–positive people is working, with an additional 2.3 million people accessing treatment in 2013, bringing the global number to 13 million, and estimated to reach almost 14 million during 2014. In sub–Saharan Africa, 76% of people on ART have viral suppression, whereby transmission is unlikely; and every 10% increase in treatment means a 1% decline in new infections. If HIV scale–up is accelerated by 2020, the HIV epidemic could end by 2030, averting 18 million new infections and 11.2 million deaths from 2013–2020. To achieve this, it is essential to: focus on under–served populations at higher risk of HIV, addressing their specific needs to ensure service access; tackle complex micro–epidemics with speedy tailored solutions; and end stigmatisation to ensure equal access to quality HIV services as both a human rights and public health imperative. (*UNAIDS*, 20 Jul 2014)

➢ UNAIDS welcomed Uganda’s Constitutional Court decision to overturn the law that allowed for 14–year jail terms for a first conviction, and life imprisonment for ‘aggravated homosexuality’. Although homosexuality remains illegal, the annulment means that gay men and men who have sex with men are more likely to seek HIV testing, prevention and treatment services. “President Yoweri Museveni has personally indicated to me that he wants Uganda to accelerate its AIDS response to ensure all people have access to life–saving services,” said Michel Sidibé, the Executive Director of UNAIDS. (*UNAIDS*, 1 Aug 2014)

## UNITED NATIONS

➢ The UN Environment Assembly (UNEA) met for the first time in Nairobi, Kenya, to discuss illegal wildlife trade, chemical waste and air pollution, and new universal development goals. In his opening message, Achim Steiner, the UN Environmental Protection (UNEP) Executive Director, said that the UNEA embodies the notion that challenges are best addressed and opportunities realised when nations and citizens join to promote economic prosperity, social equity and environmental sustainability. The UNEP also launched a report on South–South trade and the green economy, which explored the growing trend of development “for the South, by the South”. It coincided with the launch of a joint UNEP–INTERPOL report that highlights the links between environmental crime insecurity. (*UN News*, 23 Jun 2014)

The UN documented more than 4000 incidents of children recruited into armed conflicts in 2013, with thousands more estimated to have joined armies and rebel groups. In its annual report on children and armed conflict, Nigeria’s extremist group Boko Haram is included on its list of eight government forces and 51 armed groups that recruit, use, kill, or commit sexual violence against children, or attack schools and hospitals – and these groups generally act with impunity. Advances by Islamic extremist groups in Iraq create dangerous conditions for children, and there is widespread use of child soldiers in South Sudan, with the outbreak of fighting in December reversing recent gains. However, one hopeful sign is Chad’s removal from the UN list as its army has implemented a joint UN action plan to protect children. (*ndtv.com*, 2 Jul 2014)

➢ The 2014 UN Millennium Development Goals (MDG) report shows progress in eradicating extreme poverty, but that 20% of people in developing countries still live on less than US$ 1.25/d. The MDG of halving global poverty by 2015 has been achieved, although progress is uneven with some regions (eg, Eastern and South Eastern Asia) meeting the target, with other regions (eg, sub–Saharan Africa, Southern Asia) lagging behind. China has made huge progress in reducing its extreme poverty rate, from 60% to 12%. However, along with India, it has the largest share of global extreme poor. Apart from India and China, high poverty rates are often found in small, fragile countries that are affected by conflict. (*CNBC*, 16 Jul 2014)

➢ Some development experts claim that the UN’s next set of development goals should comprise five discrete, quantitative, achievable goals. The Copenhagen Consensus Center think–tank has devised a methodology to assess how to spend finite resources on global development, creating a cost–benefit analysis for proposals and ranking them by their effectiveness. The UN’s Open Working Group used this method on their development targets to assess their value for money. The targets that provide “phenomenal” value for money (benefits are 15 times greater than costs) include universal health coverage, access to education, free trade, and R&D on communicable disease treatments that affect developing countries. Poor value targets (benefits are uncertain, or less than cost) include widening access to higher education and investment in renewable energy. (*WSJ*, 25 Jul 2014)

➢ World leaders were joined by business, finance and civil society leaders at the UN September 2014 Climate Summit, aiming to create political momentum for a universal climate agreement in Paris in 2015, and to galvanise action towards reducing emissions and increasing resilience to climate change. The summit committed to limiting global temperature rises to 2°C, within the context of eradicating extreme poverty and promoting sustainable development. Outlining the Summit’s long–term vision, the Chair, the UN Secretary–General Mr Ban Ki–moon, announced that without timely and significant cuts in emissions it will not be possible to limit temperature rises to 2°C. He called for maintaining the Summit’s spirit of commitment and action, and for looking back on the Summit as the point where the human race decided to make the world sustainable, safe and prosperous for future generations. (*UN*, 23 Sep 2014)

## UNICEF

➢ In efforts to prevent the spread of polio and other diseases amongst children displaced by violence into the Kurdish Region of Iraq, UNICEF and the Kurdistan Regional Government have agreed to extend immunisation to host populations, displacement camps and border crossings, as an estimated 300 000 people have fled the ongoing conflict. This is heightened by the re–appearance of polio in Iraq after 14 years’ absence, where poor routine immunisation and problems in reaching children in conflict zones makes the region vulnerable to a large outbreak. UNICEF calls for extending immunisation services beyond the Syrian refugee camps, and for locating polio immunisation teams at refugee transit points. The government agreed to support vaccinations at key points, followed by a catch–up polio campaign targeting more than 700 000 children aged under 5 years. (*UNICEF*, 19 Jun 2014)

➢ According to UNICEF’s new population estimates, by the end of the 21st century 40% of all people and nearly 50% of all children will be African, heralding a radical demographic shift. Whilst other regions are seeing slower increases or declines in births, 1.8 billion babies could be born in Africa over the next 35 years, and the total population could quadruple to 4.2 billion by 2100. Africa could either reap a demographic dividend from a large workforce with relatively few dependents, or efforts to eliminate poverty by economic growth could be undermined by the population burden. Africa’s life expectancy is also increasing, and is becoming more urbanised and crowded. These changes are often happening in the poorest and most fragile countries, and UNICEF calls for more action to deal with the challenges of African’s growing population. (*The Globe and Mail*, 12 Aug 2014)

➢ Globally, more than 700 million women were younger than 18 years on marriage, with 1–in–3 married before their 15th birthday, according to a UNICEF survey. On current trends this could reach 1 billion by 2050. Although relative numbers have fallen, increasing population growth in countries where it is practised means numbers are constant. The same survey found that 130 million women and girls have experienced female genital mutilation (FGM). Although the prevalence of FGM has fallen sharply, an additional 63 million girls could face FGM by 2050. Girls who marry under 18 are less likely to remain in school, have a higher risk of domestic violence and death from pregnancy and childbirth complications. FGM risks excessive bleeding, infection, infertility and death. UNICEF’s Chief Executive, Anthony Lake, calls for increased efforts to break the cycles of FGM and child marriage. (*The Guardian*, 22 Jul 2014)

➢ The UNICEF report *Hidden in Plain Sight* uncovers a ‘shocking prevalence’ of emotional, physical and sexual violence against children – often in their own communities, homes and schools, and much going unreported. It found that homicide is the leading cause of death amongst males aged 10–19 years in many Latin American countries, and Nigeria had the highest number of young murder victims – almost 13 000 in 2012. UNICEF has urged governments to do more to protect the rights of children, and also noted that 1 in 10 girls have been raped or sexually assaulted. It blames social attitudes on gender and child–rearing as being partially–responsible for the figures. “Too many victims, perpetrators and bystanders see it as normal, and when violence goes unnoticed and unreported, we fuel the belief among children that it is normal”, says UNICEF’s Deputy Director, Geeta Rao Gupta. (*Deutsche Welle*, 5 Sep 2014)

➢ Ghana launched its National Newborn Strategy and Action Plan to guide newborn delivery and care. The Minister of Health, Dr Agyemang–Mensah, said that the country’s statistics on newborn mortality were a “serious affront”. As a result, Ghana has made little progress in reducing newborn mortality, and is unlikely to meet Millennium Development Goal on reducing under–5 mortality rates. Each year, 30 000 newborn babies die in Ghana – 71% of which is preventable if care during and after birth is available. Mrs Susan Ngongi, the UNICEF Country Representative, welcomed these efforts to reduce newborn deaths. (*ghanaweb.com*, 31 Jul 2014)

## WHO

➢ The 67th World Health Assembly (WHA) saw a record number of agenda items, documents and resolutions, and closed with the adoption of more than 20 resolutions on public health issues of global importance. They focused on: antimicrobial drug resistance; implementation of the International Health Regulations; health impact of exposure to mercury and mercury compounds; the global challenge of violence, particularly against women and girls; renewed commitments towards universal health coverage; financing and co–ordinating health research and development; access to essential medicines; regulatory system strengthening; health intervention and technology; health in the post–2015 agenda; and newborn health. The agenda reflected both the growing complexity of health issues, and the drive to address them. (*WHO*, 24 May 2014)

➢ WHO data shows that 38 million people die from preventable and treatable chronic illnesses (eg, diabetes, heart disease) each year, and nearly half die prematurely before the age of 70 years. The majority are in developing countries. Deaths from these illnesses have increased since 2000, especially in South East Asia and the Western Pacific, but Africa will see the largest increases by 2020. Progress in tackling this has been inadequate and uneven, although more than 50% of governments have signed up to a WHO action plan to reduce these deaths by 25% by 2025. WHO notes that falling rates of childhood obesity – an indicator for these diseases – would indicate progress, and that prevention, early diagnosis and treatment are important in reducing these deaths. (*Voice of America*, 10 Jul 2014)

➢ According to the WHO, the current Ebola outbreak is the “most severe acute health emergency in modern times”, and proves that the world is not prepared to respond adequately to severe public health emergencies. The WHO’s director–general, Margaret Chan, said that the disease is now “rising exponentially” in Guinea, Liberia and Sierra Leone, and could threaten the very survival of societies and governments in very poor countries. She added that she had never seen an infectious disease contribute so strongly to potential state failure. (*Telegraph UK*, 13 Oct 2014)

➢ According to WHO figures, there are more than 10 000 cases of Ebola globally in the largest outbreak of the disease, mainly in Guinea, Liberia and Sierra Leone. There are a suspected 4665 cases and 2705 deaths in Liberia, 3896 cases and 1281 deaths in Sierra Leone, and 1553 cases and 926 deaths in Guinea. Mali had one reported Ebola case, which resulted in death, Nigeria had 20 cases and eight deaths, Senegal and Spain had one case and no deaths, and the US had four cases and one death. Approximately 450 health workers are likely to have been infected with Ebola, although a large number is unrelated to caring and treating Ebola patients. (*UPI*, 25 Oct 2014)

➢ The WHO confirmed that a bubonic plague outbreak in Madagascar has killed 40 people and infected 80 others, warning of the dangers of a rapid spread of the disease, especially in the densely–populated capital city, Antananarivio. The bubonic plague is normally spread by bites from an infected flea, and there are high resistance levels to insecticide amongst fleas. However, 2% of cases in Madagascar are the more dangerous pneumonic form, which is spread person–to–person by coughing, and a task–force has been convened to manage the outbreak. (*BBC*, 21 Nov 2014)

